# Functional outcomes of ischemic stroke patients aged over 80 years treated with acute revascularization therapy according to pre-morbid disability: a PARADISE study

**DOI:** 10.3389/fneur.2023.1186288

**Published:** 2023-06-22

**Authors:** Ségolène Ruel, Laura Baptiste, Gauthier Duloquin, Yannick Béjot

**Affiliations:** Dijon Stroke Registry, Department of Neurology, University Hospital of Dijon, University of Burgundy, Dijon, France

**Keywords:** ischemic stroke, older patients, thrombolysis, mechanical thrombectomy, outcome, disability

## Abstract

**Introduction:**

Aging population leads to changes in the profile of patients with acute ischemic stroke (IS), and older adults were largely excluded from randomized clinical trials of acute revascularization therapy. This study aimed to assess functional outcomes of treated IS patients > 80 years old according to prior disability and identify associated factors.

**Methods:**

Consecutively older patients with acute IS treated with either IV thrombolysis and/or mechanical thrombectomy were enrolled between 2016 and 2019. Pre-morbid disability was assessed using the modified Rankin Scale (mRS) score, and patients were classified as being independent (mRS score, 0–2) or having pre-existing disability (mRS score, 3–5). A multivariable logistic regression analysis was performed to assess factors associated with a poor functional outcome (mRS score > 3) at 3 and 12 months in each group of patients.

**Results:**

Among 300 included patients (mean age: 86.3 ± 4.6 years, 63% women, median NIHSS score: 14, IQR: 8–19), 100 had a pre-existing disability. In patients with a pre-morbid mRS score of 0–2, 51% had mRS >3 including 33% of deaths at 3 months. At 12 months, 50% had a poor outcome including 39% of deaths. In patients with a pre-morbid mRS score of 3–5, 71% had a poor outcome at 3 months including 43% of deaths, and at 12 months, 76% had mRS >3 including 52% of deaths. In multivariable models, the NIHSS score at 24 h was independently associated with poor outcomes at 3 and 12 months in both patients with (OR = 1.32; 95% CI: 1.16–1.51, *p* < 0.001 for 12 months outcome) or without (OR = 1.31; 95% CI: 1.19–1.44, *p* < 0.001 for 12 months outcome) pre-morbid disability.

**Conclusion:**

Although a large proportion of older patients with a pre-existing disability had a poor functional outcome, they did not differ from their non-impaired counterparts regarding prognostic factors. This means that there were no factors in our study that would help clinicians identify patients at risk of poor functional outcomes after revascularization therapy among those with prior disability. Further studies are needed to better understand the post-stroke trajectory of older IS patients with a pre-morbid disability.

## 1. Introduction

Stroke is the second leading cause of both disability and death worldwide (1). and despite declining or stable incidence rates in Western countries, the absolute number of patients suffering a cerebrovascular event each year has increased by 70% over the last 30 years (2). The ongoing aging population largely contributed to this trend (2, 3), and epidemiological projections indicate that this phenomenon will magnify since the number of people aged over 80 years is expected to double in the US and Europe by 2050, thus leading to a dramatic expected rise in stroke cases (2). Consequently, the management of older patients represents a key issue in stroke medicine.

Ischemic stroke (IS) has benefited from major therapeutic advances in acute recanalization therapy including intravenous thrombolysis (IVT) and mechanical thrombectomy (MT). Older patients have been poorly represented in randomized clinical trials while they have a less favorable functional outcome after stroke (4). Nevertheless, they seem to benefit as much as young patients from both IVT (5, 6) and MT (7–10) regarding 3- and 6-month outcomes (6–9) but few studies described long-term outcomes (11). In addition, patients with a prior-to-stroke disability defined as a pre-morbid modified Rankin scale (mRS) score (12) of either >2 or >3 were excluded from most trials of acute IS revascularization therapy, regardless of age. Considering that more than one-third of older stroke patients have a pre-morbid disability, such an exclusion criterion is questionable in terms of the reproducibility of the results of these trials in patients managed in clinical routine (13, 14).

Therefore, this study aimed to assess early and long-term functional outcomes of IS patients > 80 years old treated with acute revascularization therapy according to prior disability and to identify their associated factors.

## 2. Methods

### 2.1. Patients and study design

**Figure 1:**
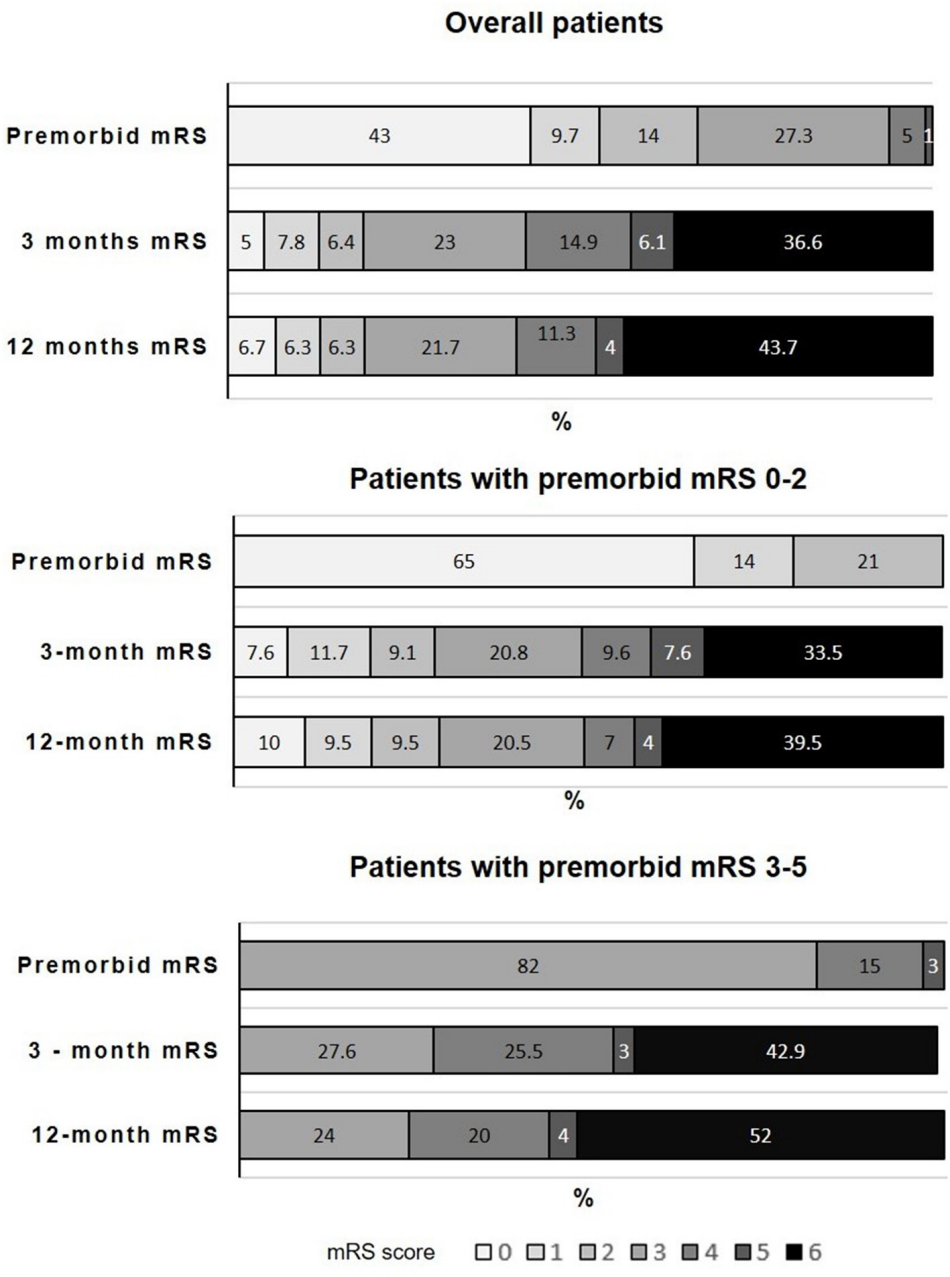
Distribution of patients according to pre-morbid 3- and 12-month mRS scores.

The Prognosis After Revascularization therapy in the Dijon Ischemic Stroke Evaluation (PARADISE) study was a single-center prospective observational cohort study conducted from January 2016 to June 2019 at the Dijon University Hospital (ClinicalTrials NCT02856074). Patients aged 18 years or older were consecutively included if they had an acute ischemic stroke (IS) treated with revascularization therapy (either IVT and/or MT). Patients received information about the study and gave their oral consent to participate in an agreement with French legislation. The study was approved by a French Ethics Committee (CPP Est I, IRB number: 2015-A01664-45). For the present study, we included and analyzed the data from all patients aged 80 years or older enrolled in the PARADISE study who achieved a complete follow-up period of 12 months (Figure 1).

### 2.2. Data collection and patient's follow-up

At inclusion, we collected sociodemographic characteristics, medical history, vascular risk factors, and pre-stroke antithrombotic treatments. Pre-morbid disability was assessed using the mRS score. The assessment was performed by senior stroke-trained neurologists who are familiar with the use of this score in routine practice and who had been previously certified due to their participation in several randomized controlled trials. Patients were classified into two groups accordingly: those with functional independence (mRS score 0–2) and those with a pre-existing mild-to-severe disability (mRS score 3–5). Prior-to-stroke cognitive function was categorized as no cognitive impairment; mild cognitive impairment (MCI), which was defined as a cognitive decline without any interference with activities of daily life; or dementia, which was defined as a cognitive decline sufficient to interfere with independence in activities of daily living. Stroke severity was measured on admission and at 24 h using the National Institutes of Health Stroke Scale (NIHSS) score. This score was prospectively reported in the medical file by a certified stroke-trained neurologist based on his clinical exam. The arterial occlusion site responsible for the acute ischemic stroke was recorded and was classified as follows: proximal occlusion when the thrombus was in the terminal intracranial internal carotid artery, the M1 and M1/M2 junction segments of the middle cerebral artery (including tandem occlusions), and the basilar artery; distal occlusion corresponding to a thrombus site in the anterior or posterior cerebral arteries, the distal segments of the middle cerebral artery, and isolated vertebral artery; and isolated occlusion of the extracranial internal carotid artery. Hemorrhagic transformation of ischemic stroke was assessed on systematic imaging performed at 24 h following admission. We considered any hemorrhagic transformation according to the NINDS criteria (15).

Patients were followed by phone contact or by a consultation at 3 and 12 months. During each visit, the functional status of patients was assessed with the mRS score. The mRS score was assessed by either a stroke neurologist if the patient was seen in consultation or by a trained clinical research assistant if the patient was contacted by phone, using a structured questionnaire. A good outcome was defined as an mRS score between 0 and 3 and a poor outcome as an mRS score between 4 and 6 (including death). We chose to consider a post-stroke mRS score of 0–3 as a favorable clinical outcome rather than an mRS score of 0–2 because of the high frequency of prior disability in patients > 80 years old due to comorbidities, and the fact that choosing an mRS score of 0–2 would have prevented the potential demonstration of a favorable effect of acute revascularization therapy in patients with a previous disability (prior mRS score, 3–5).

### 2.3. Statistical analyses

Categorical variables were described as frequencies and quantitative variables as means with standard deviations (SD) or medians with interquartile range (IQR). Characteristics of patients according to their pre-stroke disability were compared using Fisher's exact test, chi-square test, Mann–Whitney test, or Wilcoxon test, when appropriate. A logistic regression analysis was conducted to assess factors associated with a poor functional outcome (defined as mRS score ≥ 4) at 3 and 12 months, in each group of patients (prior-to-stroke functional independence and pre-existing disability). In multivariable models, we introduced variables with a *p*-value of < 0.20 in the univariable analysis. Because of collinearity with pre-morbid mRS, pre-stroke cognitive function was not considered in multivariable models. Statistics were performed using SAS^®^ 9.3 software (Cary, NC, USA).

## 3. Results

Between January 2016 and June 2019, 329 IS patients aged 80 years or older treated with acute revascularization therapy were included. Information about functional outcomes at 1 year was available for 300 patients (mean age: 86.3 ± 4.6 years, 63% women, median NIHSS score: 14, IQR: 8–19). Among these patients, 200 had a pre-morbid mRS score of 0–2 and 100 had a pre-existing disability.

The characteristics of patients are shown in Table 1. Patients with a pre-existing disability were older, had a more frequent history of stroke, MCI, or dementia, and had less frequent hypercholesterolemia than those with a pre-morbid mRS score of 0–2. In contrast, they did not differ regarding other vascular risk factors and prior antithrombotic treatments, stroke severity, and occlusion site. The distribution of the type of revascularization therapy was similar between both groups with approximately half of patients treated with IVT alone, one-third with MT alone, and the remaining 15% with bridging therapy. Timing of acute revascularization therapy, median NIHSS score at 24 h, and the proportion of patients who had any hemorrhagic transformation of IS on imaging at 24 h did not differ between the groups.

**Table 1:** Characteristics of patients according to a pre-morbid mRS score.

	**Overall patients (*****N*** = **300)**		**Patients with premorbid mRS 0–2 (*****N*** = **200)**		**Patients with premorbid mRS 3-5 (*****N*** = **100)**		***p***
	***n*** **%**		***n*** **%**		***n*** **%**		
**Age, y**							
Mean ± SD	86.3 ± 4.6		85.5 ± 4.2		87.9 ± 4.9		**< 0.001**
Median (IQR)	86 (83–89)		85 (82–88)		87 (84.5–91.5)		
**Sex**							
Male	111	37	77	38.5	34	34	0.447
**Medical history**							
Hypertension	224	75.4	147	73.87	77	78.57	0.376
Myocardial infarction	29	9.9	15	7.58	14	14.58	0.059
Atrial fibrillation	169	57.1	105	53.3	64	64.65	0.063
Previous stroke	44	14.9	22	11.2	22	22.45	**0.010**
Previous TIA	20	6.8	10	5.1	10	10.3	0.096
Diabetes mellitus	52	17.7	35	17.77	17	17.53	0.959
Hypercholesterolemia	107	36.3	82	41.84	25	25.25	**0.005**
Sleep Apnea Syndrome	29	9.8	18	9.05	11	11.34	0.533
Cognitive impairment	70	27.1	25	14.79	45	50.56	**< 0.001**
Mild cognitive impairment	46	17.8	23	13.61	23	25.84	**0.013**
Dementia	24	9.3	2	1.18	22	24.72	**< 0.001**
Current or past smoking	53	19.2	37	20.22	16	17.2	0.548
**Prestroke antithrombotic therapy**							
Antiplatelet agent	106	36.2	71	36.41	35	35.71	0.907
Anticoagulant	57	19.5	36	18.46	21	21.43	0.545
**Initial clinical features**							
Wake-up stroke	44	14.7	30	15	14	14	0.817
NIHSS score at onset							0.158
Mean ± SD	14 ± 7		13 ± 7		15 ± 7		
Median (IQR)	14 (8–19)		14 (7–19)		15 (8–21)		
**Imaging data**							
CT-scan	246	82	163	81.5	83	83	0.75
MRI	105	35	72	36	33	33	0.08
Occlusion site							0.488
Proximal	154	51.3	107	53.5	47	47	
Distal	56 13	18.7	34	17	22	22	
Extracranial internal carotid artery alone		4.3	7	3.5	6	6	
**Acute revascularization therapy**							
IV thrombolysis only	145	48.3	95	47.5	50	50	0.683
Mechanical thrombectomy only	110	36.7	73 32	36.5	37	37	0.932
Combined treatment	45	15		16	13	13	0.493
Time to IV thrombolysis, min							0.830
Mean ± SD	189 ± 60		190 ± 66		187 ± 45		
Median (IQR)	180 (150–217)		180 (145–225)		178 (155–208)		
Time to thrombectomy, min							0.959
Mean ± SD	376 ± 353		381 ± 379		367 ±298		
Median (IQR)	293 (177–410)		298 (167–417)		262 (185–405)		
**24-h evolution**							
NIHSS score at 24 h							0.155
Mean ± SD	11 ± 8		10 ± 8		11 ± 8		
Median (IQR)	9 (3–16)		8 (3–17)		10 (5–16)		
Hemorrhagic transformation at 24 h	55	20.6	36	20.4	19	20.88	0.935

The bold values indicate the statistically significant values.

Globally, 58% of overall patients had a poor outcome (defined as an mRS score >3) at 3 months including 37% of dead patients (Table 2; Figure 1). At 12 months, 59% of patients had a poor outcome including 44% who died. The distribution of mRS score at 12 months according to pre-stroke functional status is shown in Table 3. In patients with a pre-morbid mRS score of 0–2, 28% maintained an mRS score of 0–2 at 3 months, and 51% had an mRS score of >3 including 33% of deaths. At 12 months, 29% had an mRS score of 0–2, and 50% had a poor outcome including 39% of deaths. In patients with a pre-morbid mRS score of 3–5, 71% had a poor outcome at 3 months including 43% of deaths. The death rate was 52% at 12 months and 76% of patients had an mRS score of >3.

**Table 2:** Distribution of an mRS score at 3 and 12 months according to a pre-morbid mRS score.

	**Overall patients (*****N*** = **300)**		**Premorbid mRS 0–2 (*****N*** = **200)**		**Premorbid mRS 3-5 (*****N*** = **100)**		***p***
	***n*** **%**		***n*** **%**		***n*** **%**		
**mRS score**						
At 3 months							< 0.001
0–2	57	19.3	56	28.4	1	1.0	
3–5	130	44.1	75	38.1	55	56.1	
6	108	36.6	66	33.5	42	42.9	
At 12 months							< 0.001
0–2	57	19	57	28.5	0	48	
3–5	111	37	63	31.5	48	52	
6	132	44	80	40	52		

**Table 3:** Distribution of an mRS score at 12 months according to the pre-morbid mRS score.

	**12 months mRS**						
**Premorbid mRS**	**mRS 0**	**mRS 1**	**mRS 2**	**mRS 3**	**mRS 4**	**mRS 5**	**mRS 6**
mRS 0 (*N* = 129)	14.7%	14%	8.5%	18.6%	5.4%	3.1%	35.6%
mRS 1 (*N* = 29)	3.5%	3.5%	17.2%	20.7%	10.3%	0%	44.8%
mRS 2 (*N* =42)	0%	0%	7.1%	26.2%	9.5%	9.5%	47.6%
mRS 3 (*N* =82)	0%	0%	0%	29.3%	19.5%	1.2%	50%
mRS 4 (*N* =15)	0%	0%	0%	0%	26.7%	20%	53.3%
mRS 5 (*N* =3)	0%	0%	0%	0%	0%	0%	100%

In patients with a pre-morbid mRS score of 0–2, only the NIHSS score at 24 h was associated with an increased risk of poor post-stroke functional outcome at 3 months in the multivariable logistic regression analysis (OR = 1.37; 95% CI: 1.23–1.53, *p* < 0.001) (Table 4). At 12 months, both NIHSS scores at 24 h (OR = 1.31; 95% CI: 1.19–1.44, *p* < 0.001) and hemorrhagic transformation (OR = 5.16; 95% CI: 1.46–18.18, *p* = 0.011) were independently associated with poor outcome (Table 5). In patients with a pre-morbid mRS score of 3–5, only the NIHSS score at 24 h was associated with a higher risk of poor outcome at both 3 months (OR = 1.26; 95% CI: 1.13–1.41, *p* < 0.001) and 12 months (OR = 1.32; 95% CI: 1.16–1.51, *p* < 0.001).

**Table 4:** Factors associated with a post-stroke mRS score ≥ 4 at 3 months in multivariable logistic regression analysis, in patients with a pre-morbid mRS score of 0–2.

	**OR**	**95% CI**	***P*-value**
Age	1.03	0.90–1.19	0.639
Atrial fibrillation	1.18	0.34–3.68	0.772
Pre-stroke cognitive status			
No cognitive impairment	Ref		
MCI	1.38	0.20–9.38	0.744
Initial MRI	2.58	0.78–8.49	0.119
Occlusion site			
Not visible	Ref		
Proximal	2.42	0.34–16.99	0.373
Distal	0.27	0.06–1.46	0.130
IV thrombolysis only	3.14	0.37–26.61	0.295
Mechanical thrombectomy only	0.94	0.17–5.06	0.942
NIHSS score at 24 h	1.37	1.23–1.53	**< 0.001**
Hemorrhagic transformation	1.41	0.41–4.84	0.583

The bold values indicate the statistically significant values.

**Table 5:** Factors associated with a post-stroke mRS score of ≥4 at 12 months in multivariable logistic regression analysis in patients with a pre-morbid mRS score of 0–2.

	**OR**	**95% CI**	***P-*value**
Age	1.05	0.92–1.19	0.480
Atrial fibrillation	1.80	0.63–5.16	0.274
Pre-stroke cognitive status			
No cognitive impairment	Ref		
MCI	3.66	0.59–22.85	0.164
Occlusion site			
Not visible	*Ref*		
Proximal	2.44	0.67–8.88	0.175
Distal	0.57	0.11–2.99	0.511
NIHSS score at 24 h	1.31	1.19–1.44	**< 0.001**
Hemorrhagic transformation	5.16	1.46–18.18	**0.011**

The bold values indicate the statistically significant values.

## 4. Discussion

Our study demonstrated that although a large proportion of older patients with a pre-existing disability had a poor functional outcome after IS treated with acute revascularization therapy, they did not differ from their non-impaired counterparts regarding prognostic factors. Hence, the NIHSS score at 24 h was associated with a 3- and 12-month disability in both groups, thus suggesting that patients' response to acute therapy appeared to be the best predictor of recovery. This means that there were no factors in our study that would help clinicians identify patients at risk of poor functional outcome after revascularization therapy among those with a prior disability, and that the unfavorable outcome of patients with a prior disability could be due to the pre-morbid status itself.

Some differences in baseline characteristics of patients according to prior disability were observed. Hence, patients with a pre-morbid disability had less often hypercholesterolemia, which may suggest a less intensive screening for this vascular risk factor in dependent patients. Furthermore, the presence of pre-existing cognitive disorders was greater in patients with a pre-morbid mRS score of >2. One of the reasons is that cognitive impairment influences mRS scoring. Therefore, it was not possible to introduce this variable in multivariate models due to some collinearity. Of note, cognitive impairment has been associated with poorer functional outcomes and higher post-stroke mortality in previous studies (16, 17). This may be explained by the clinical profile of these patients who are older, have high-risk comorbidities, have a more severe initial clinical presentation, and are less often hospitalized in intensive care units (18). Although the pathophysiology is not fully understood, the vascular or degenerative brain damage that contributes to cognitive impairment seems to make the brain more vulnerable to acute ischemia (19). In addition, the presence of cognitive disorders may limit post-stroke rehabilitation, thus contributing to a worse prognosis.

In our older population with prior disability, access to recanalization therapy was associated with a return to previous autonomy (mRS between 3 and 5) in 55% of cases at 3 months and 48% at 1 year. In detail, 33% of patients with an initial mRS score of 3 returned to this score at 3 months and almost 30% at 1 year. Corresponding rates in those with a pre-morbid mRS score of 4 were 46 and 26%, respectively. All severely disabled patients with a pre-morbid mRS score of 5 died at 3 months. These results are consistent with the limited data available in the literature. In the SITS-EAST study, almost one in three patients returned to a previous mRS score at 3 months after thrombolysis, in both groups (20). Even though pre-stroke disability was associated with higher mortality and worse neurological functional outcomes at 3 months, the use of IV thrombolysis appeared to be of benefit to some previously dependent patients (21). A recent meta-analysis of five observational studies of MT concluded a “good clinical outcome,” which was defined as an mRS score of 0–2 or a return to the pre-stroke mRS score in 27% of patients at 3 months (10). However, the analysis was not restricted to older patients, thus explaining that the mean age was lower than in our study. In addition, no long-term outcome was reported in any study of revascularization.

The NIHSS score at 24 h appeared as a robust factor associated with post-stroke outcomes in our study, whatever the prior functional status of patients. This score reflects in part the effectiveness of revascularization treatment, and it has been demonstrated that it has a good correlation with the mRS score at 3 and 12 months (22). Therefore, it has recently been suggested as an excellent surrogate criterion for future studies (23) although it is limited by the fact that it does not evaluate the consequences on the autonomy of patients in their daily life.

No additional factors were associated with poor outcomes in patients with prior disability. In other words, our study did not reveal factors that could help clinicians identify a subgroup of patients for whom acute revascularization treatment would be particularly deleterious. However, as treatment indications were made by stroke-trained neurologists in a tertiary center (either locally or by telemedicine procedure), we can hypothesize that there was a selection of patients eligible for acute therapy. Hence, some patients may have been excluded based on medical judgment because of comorbidities including frailty. In agreement with this hypothesis, we observed lower rates of prior dementia than reported in population-based studies (4, 19), suggesting that non-negligible severely cognitively impaired patients were not considered for treatment. However, whether these patients may have a long-term benefit from revascularization therapy remains poorly documented since they have been largely excluded from randomized clinical trials. In our study, patients were selected by stroke neurologists, which consider potential benefit of recanalization therapy, despite prior disability. For instance, patients with a prior motor impairment who had acute stroke IS with severe aphasia may have been included with the expectation that they would recover from speech impairment, thereby reducing the risk of further disability.

Growing evidence from the literature suggests that age should not be an isolated criterion for excluding revascularization treatment (24, 25). However, it seems necessary to identify reliable factors to help clinicians in the decision-making process. For instance, recent observational studies have shown an interest in considering the volume of the ischemic core on brain CT-scan in elderly patients, particularly those over 80 years old (26, 27).

Regarding safety, in our study, hemorrhagic transformation occurred in 20% of patients, and the proportion did not differ between patients with and without pre-stroke disability which is in agreement with the literature (20, 21, 28).

Our study has several limitations. The restricted sample size made it difficult to conduct subgroup analyses, and larger cohorts were required. In addition, reasons for pre-existing disabilitys were not registered, and we measured it based on the mRS score, which incompletely captures the complexity of measuring pre-stroke dependency (29) and does not explore frailty. Furthermore, we did not include untreated patients. Therefore, we were not able to assess the contribution of acute revascularization therapy on post-stroke disability. Although we observed that one-quarter to a third of patients with prior disability returned to their previous mRS score, our study was not designed to comprehensively explore the issue of the futility of revascularization therapy in them.

To conclude, although older patients with a pre-existing disability had a poor functional outcome after IS treated with acute revascularization therapy, we did not identify other deleterious factors than the NIHSS score at 24 h. Further studies are needed to better understand the post-stroke trajectory of older IS patients with a pre-morbid disability after acute revascularization therapy.

## Data availability statement

The raw data supporting the conclusions of this article will be made available by the authors, without undue reservation.

## Ethics statement

The studies involving human participants were reviewed and approved by French Ethics Committee (CPP Est I, IRB number: 2015-A01664-45). Written informed consent for participation was not required for this study in accordance with the national legislation and the institutional requirements.

## Author contributions

SR: study concept and design, acquisition, analysis and interpretation of data, reviewing arterial imaging, and drafting and revising the manuscript for content. LB and GD: acquisition of data and critical revision of the manuscript for intellectual content. YB: study concept and design, acquisition, analysis and interpretation of data, study supervision, obtaining funding, and drafting and revising the manuscript for content. All the authors contributed to the article and approved the submitted version.
